# Variantia Rerum

**Published:** 1837

**Authors:** 


### Art. IX.—Variantia Rerum.

*Recognitions of the Cincinnati College.*—Since the six letters of recognition of the Medical Department of the Cincinnati College were published in the appendix to our last number, we have received another, from Prof. Geddings, Dean of the Faculty of the Medical College of South Carolina. Our Transylvania friends have not yet got through with the consideration of our case; but while they place us before the profession, *publicly*, as an institution, which, in their opinion, is unworthy of being recognised, some of them are giving notice *privately* (as we understand) to pupils of our last year's class, that they may graduate next spring, if they will come to Lexington this fall!

*New Appointments.*—Dr. Griffith, of the University of Maryland, has been called to the chair of Theory and Practice, Obstetrics, and Medical Jurisprudence in the University of Virginia; and Dr. Cabell, of Virgina, not hitherto a teacher, has been appointed professor of Anatomy, Physiology and Surgery, in the same school.

Drs. Howard, Dorsey, Finley, Fisher and Hughes, all of the State of Maryland, have been appointed into the medical department of the University of that State. The professorship of Surgery remains unfilled. Thus this ancient institution will have an entirely new Faculty.

The professors of the Medical College of Georgia, for the next session, are Drs. Dugas, Cunningham, Eve, Antony, P. F. Eve, Davis, Newton and Ford—*eight* in number, with a session of *six* months. We say, well done, Georgia! If those who manage all our schools had a little more of the enlightened and comprehensive spirit, of which these improvements are the offspring, the American profession would soon begin to assume a nobler aspect.

*Epidemic Cholera.*—We have not heard of a case of this disease in any part of the United States. Cincinnati, throughout the summer, has presented no tendency to it; and, indeed, has enjoyed remarkable health.

*Yellow Fever in New-Orleans.*—It appears that this disease is prevailing with a decided character of mortality in that city. We hope some of our friends will favor us with an account of it for the next number of the Journal. We should like, above all things, to have a full and candid statement of the comparative efficacy of the old mercurial, and the new topical bleeding and gum water, practice.

*Lectures on Meteorology.*—Mr. Espy, of Philadelphia, one of the most ingenious meteorologists, of the age, has lately made a visit to Columbus, in this State, and delivered a short series of lectures on the science which he has cultivated with so much success. Why did he not come to Cincinnati?

*Electro-Magnetic power.*—Among the recent inventions for the application of this power to the propulsion of machinery, is one by Mr. Abel Shawk, an ingenious artist of this city. Competent judges regard it as of high promise; and the opinion seems to be daily acquiring strength, that electro-magnetism will at length be applied to many valuable purposes in the arts.

*Bibliographical Record.*—In consequence of the severe and protracted illness of Prof. Gross, (from which he is now recovering, Sept. 18) no bibliographical record will be appended to this number; that gentleman having the immediate charge of this subject. We take this occasion to correct a small error in the record for July. Curling's Treatise on Tetanus is there mentioned as a republication in Dunglison's Medical Library—it should have been Bell's. A further consequence of Professor Gross's illness, is the omission, in this number, of a review of Bushe on the Rectum, promised in the same record.

*Times of publication.*—As we shall henceforth finish the printing of every number at least a fortnight before the appointed time for issuing it, that is, by the middle of the months of December, March, June, and September, we must beg of our correspondents to be prompt in sending forward their communications. D.

